# A New Regression Model for Depression Severity Prediction Based on Correlation among Audio Features Using a Graph Convolutional Neural Network

**DOI:** 10.3390/diagnostics13040727

**Published:** 2023-02-14

**Authors:** Momoko Ishimaru, Yoshifumi Okada, Ryunosuke Uchiyama, Ryo Horiguchi, Itsuki Toyoshima

**Affiliations:** 1 Division of Information and Electronic Engineering, Muroran Institute of Technology, 27-1, Mizumoto-cho, Muroran 050-8585, Japan; 2 College of Information and Systems, Muroran Institute of Technology, 27-1, Mizumoto-cho, Muroran 050-8585, Japan

**Keywords:** audio feature, depression, regression model, correlation, graph convolutional neural network

## Abstract

Recent studies have revealed mutually correlated audio features in the voices of depressed patients. Thus, the voices of these patients can be characterized based on the combinatorial relationships among the audio features. To date, many deep learning–based methods have been proposed to predict the depression severity using audio data. However, existing methods have assumed that the individual audio features are independent. Hence, in this paper, we propose a new deep learning–based regression model that allows for the prediction of depression severity on the basis of the correlation among audio features. The proposed model was developed using a graph convolutional neural network. This model trains the voice characteristics using graph-structured data generated to express the correlation among audio features. We conducted prediction experiments on depression severity using the DAIC-WOZ dataset employed in several previous studies. The experimental results showed that the proposed model achieved a root mean square error (RMSE) of 2.15, a mean absolute error (MAE) of 1.25, and a symmetric mean absolute percentage error of 50.96%. Notably, RMSE and MAE significantly outperformed the existing state-of-the-art prediction methods. From these results, we conclude that the proposed model can be a promising tool for depression diagnosis.

## 1. Introduction

Depression is a psychiatric disorder that can be attributed to the complex interaction of psychological and social factors. According to the World Health Organization, approximately 280 million people suffer from depression worldwide [1]. The symptoms range from long-term depressed mood or loss of interest to disrupted sleep and eating disorders, and in the worst case, depression may lead to suicide [2]. Because objective and quantitative diagnostic criteria have not yet been established, the diagnosis of depression is based on the subjective judgment of the physician. Therefore, the diagnosis of depression is often delayed or missed [3,4]. Depression is treated differently depending on its severity [5]; hence, appropriate and rapid identification of its severity is crucial for deciding on an appropriate treatment plan.

In recent years, many studies have proposed methods for predicting depression severity using deep learning algorithms to support physician diagnosis. These studies used modality data, such as audio data [6,7,8,9,10,11,12,13,14], facial expression data [11,14,15], and text data [11,14], as training data and constructed regression models based on neural networks to predict depression severity. In particular, unique audio features (biomarkers) have been reported in the voices of depressed patients [16,17]; accordingly, many audio-based depression severity prediction methods have been proposed [6,7,8,9,10,12,13]. Furthermore, audio features that show a mutual correlation among the voices of depressed patients have been detected [18]. Thus, the voices of depressed patients can be characterized based on the combinatorial relationships among multiple audio features, which is an important aspect that must be considered for accurately predicting depression severity. However, existing studies have assumed that the individual audio features are independent; thus, they have not constructed models based on the relationships among these features.

The objective of this study is to test the hypothesis that using relationships among audio features is effective for predicting the severity of depression. Hence, in this paper, we propose a new regression model that predicts depression severity on the basis of the correlation among audio features. This model was constructed using a graph convolutional neural network (GCNN) [19], a deep learning algorithm. A GCNN represents the relationship among audio features as graph-structured data, which can be used to extract the voice characteristics using a convolutional neural network (CNN) [20]. The two main contributions of this study are as follows: (1) presenting a new method using a GCNN to predict depression severity on the basis of the relationship among audio features; and (2) demonstrating better prediction performance than existing state-of-the-art methods.

## 2. Materials and Methods

As discussed in Section 1, the voices of depressed patients are distinguished based on combinatorial correlations among multiple audio features [18]. Hence, when learning the voice characteristics of depressed individuals, the correlations among audio features should be considered. Consequently, we employed a GCNN, which enables deep learning based on correlations among audio features. This is a novel approach that has yet to be explored in prior studies. The following sections describe the details of the proposed model.

### 2.1. Datasets

In this study, we used the DAIC-WOZ dataset [21], which is widely used for performance evaluation in depression severity prediction via machine learning. This dataset contains audio data collected from the responses of 189 subjects (102 males and 87 females) during interviews. The subjects were assigned scores on the basis of a depression rating scale, PHQ-8 [22]. The audio data are vectors comprising 74 audio features which are generated every 10 milliseconds from the voiced sound of each subject’s speech using the opensource program COVAREP [23]. We eliminated nine audio features, which were included in “voicing or not (VUV),” “detecting creaky voice (creak),” and “harmonic model phase distortion mean (HMPDM0-6),” from the feature vectors. This is owing to the fact that the value variances of these features were approximately zero, and the correlation coefficients could not be calculated correctly in the generation of similarity graphs in Section 2.3.1. The remaining 65 audio features were adjusted to have a zero median and a one quartile range. Finally, 6,639,782 65-dimensional feature vectors were created. The obtained feature vectors were employed as training and testing data for the proposed model.

### 2.2. Definition of Depression Severity

Table 1 shows the correspondence between the PHQ-8 score and severity level [22]. The PHQ-8 score takes integer values from 0 to 24 and is used to determine the depression severity level for each subject. Herein, following previous studies [11,12,13,14], we defined the depression severity assigned to each feature vector as the PHQ-8 score value.

### 2.3. Model Construction

#### 2.3.1. Creation of Similarity Graphs

To predict the severity of depression according to the correlation among audio features, we created similarity graphs for the audio features showing mutual correlation using the feature vectors created in Section 2.1. The similarity graphs were created via the following procedure. First, the similarities among all of the audio features were estimated using the feature vectors for training the model. Following the literature [19], we employed the absolute value of the correlation coefficient as the similarity between audio features. Next, for each audio feature, *n* audio features (referred to as neighborhoods) were selected in descending order of similarity. Herein, *n* was set to nine. Subsequently, we represented each audio feature and its neighborhood as nodes and created similarity graphs by connecting these nodes by edges (note that neighborhoods were not connected to each other). Finally, we generated 65 similarity graphs for the 65 audio features. These graphs were used for model construction and depression severity prediction.

#### 2.3.2. Training and Prediction by GCNN

**Figure 1:**
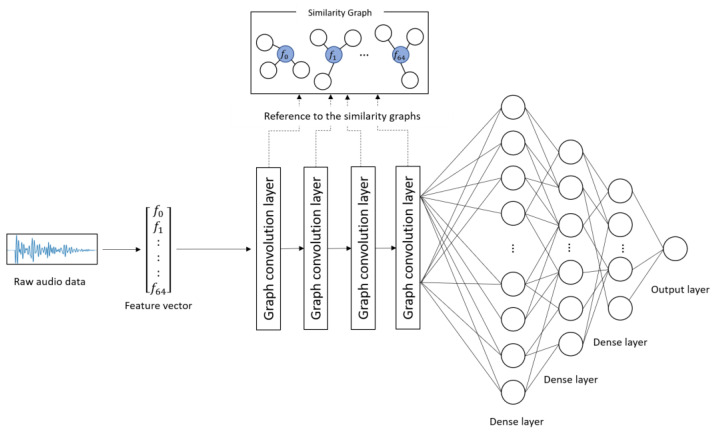
Architecture of the proposed model.

Figure 1 and Table 2 show the architecture and details of the proposed model, respectively. The proposed model learns the characteristics of the subject’s voice at each severity level on the basis of the combinations of correlated audio features in the feature vectors. The input to the model was the 65-dimentional feature vectors converted from the audio data. First, convolution was performed on each audio feature included in the similarity graphs through four graph convolution layers. This process corresponds to the convolution operation on image data using a filter matrix in CNN [20]. Subsequently, the predicted score was obtained from the output layer through three dense layers. In this study, the categorical cross-entropy error was used as a loss function, and Adam [24] was used as an optimization function. The weight parameters of the network were updated on the basis of the backpropagation algorithm by comparing the ground truth and the predicted score.

The procedure for predicting depression severity was as follows. First, a raw audio sample was converted into a 65-dimensional feature vector and input into the model. The feature vector was fed into the graph convolution layers and dense layers, and a depression severity score was predicted by the output layer.

## 3. Experimental Results

### 3.1. Experimental Method

On the basis of the feature vectors presented in Section 2.1, data were randomly selected and categorized as follows: 80% for training, 10% for validation, and 10% for testing. The prediction accuracy was evaluated using three indices, root mean square error (RMSE), mean absolute error (MAE), and symmetric mean absolute percentage error (SMAPE) as follows:

(1) $$\mathrm{RMSE}=\sqrt{\frac{1}{n}\overset{n}{\underset{i=1}{\sum }}{\left(\hat{y_{i}}-y_{i}\right)}^{2}},$$

(2) $$\mathrm{MAE}=\frac{1}{n}\overset{n}{\underset{i=1}{\sum }}\left|\hat{y_{i}}-y_{i}\right|,$$

(3) $$\mathrm{SMAPE}=\frac{100}{n}\overset{n}{\underset{i=1}{\sum }}\frac{\left|\hat{y_{i}}-y_{i}\right|}{\left(\left|\hat{y_{i}}\right|+\left|y_{i}\right|\right)/2},$$

where $\hat{y_{i}}$, $y_{i}$, and n indicate the predicted score output from the model, actual severity score, and the number of test data, respectively.

### 3.2. Results of Prediction Experiments

Table 3 shows the scores for each evaluation index obtained through the prediction experiments and a comparison with the existing state-of-the-art studies. Similar to our study, these studies were conducted using the DAIC-WOZ dataset. A, V, T, and A + V + T in the modality column represent audio modality, visual modality, text modality, and multimodality, respectively. As for SMAPE, only the value of the proposed model is shown in this table because it has not been shown in the other studies. Yang et al. [11] and Fang et al. [14] employed a multimodal model; hence, the findings for the respective modalities and the multimodality are shown in Table 3. As can be seen from Table 3, our model showed an RMSE and MAE of 2.15 and 1.25, respectively. These errors are considerably small compared with those of the other methods. This indicates that the proposed model can predict depression severity with higher accuracy than those methods. SMAPE takes values between 0 and 200%. Our model showed a SMAPE of 50.96%.

**Figure 2:**
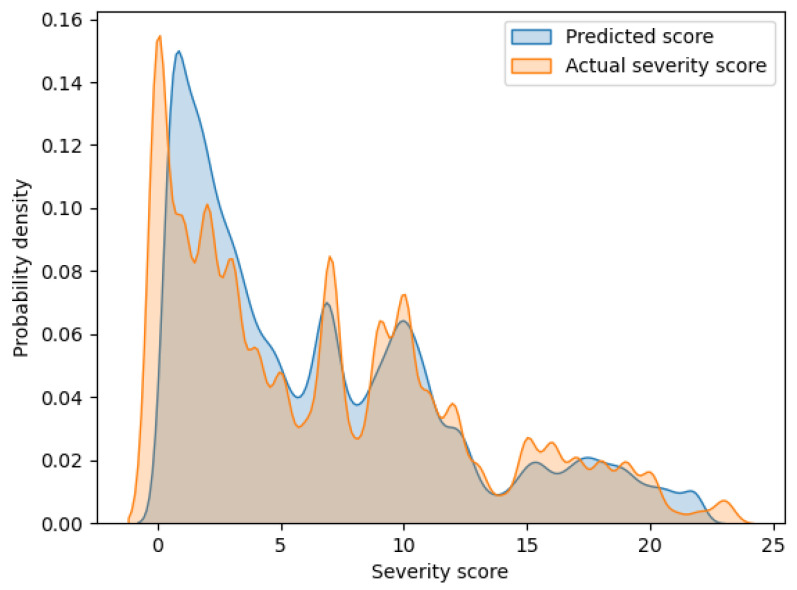
Probability density functions of the predicted scores and actual severity scores.

Figure 2 shows an overlaid graph of the probability density functions of the predicted scores and actual severity scores calculated via kernel density estimation. The horizontal and vertical axes of the graph indicate the severity score and probability density, respectively. In this graph, these two curves have a large overlap. Hence, it can be seen that the proposed model can output highly accurate prediction values.

## 4. Discussion

Yang et al. [11] introduced a multimodal model based on audio, visual, and text modalities. Their results showed that the text modality performed best, with an RMSE and MAE of 4.38 and 3.64, respectively, and the audio modality exhibited an RMSE and MAE of 5.63 and 4.85, respectively. They also focused on improving the audio modality, achieving an RMSE and MAE of 5.52 and 4.63, respectively [12]. The audio modality model proposed by Lu et al. [13] exhibited an RMSE and MAE of 5.37 and 4.48, respectively. Fang et al. [14] suggested a multimodal model that incorporated audio, vision, and text modalities. Their results showed that the multimodality performed best, with an RMSE and MAE of 3.68 and 3.18, respectively, and the audio modality exhibited an RMSE and MAE of 6.13 and 5.21, respectively.

Our proposed model outperformed the aforementioned models, with an RMSE and MAE of 2.15 and 1.25, respectively. These findings demonstrate the efficacy of predicting depression severity using correlations among several audio features based on GCNN. Another reason for the superior prediction performance of the proposed model may be the number of training data. The existing methods, in which the same dataset as our study was used, used at most 42,000 training data. Conversely, in this study, we used a large amount of training data (i.e., >6.6 million), which were obtained in a considerably short window width (10 milliseconds). As pointed out in the literature [12], a large amount of training data is required to improve prediction accuracy.

Notably, the prediction errors of the proposed model were considerably smaller than those of the other models (Table 3). The proposed model exhibited an RMSE of 2.15, while the best RMSE among the existing models is 3.68. Moreover, the proposed model exhibited an MAE of 1.25, while the best MAE among the existing models is 3.18. As explained in Section 2.2, the severity level is determined by the severity score based on PHQ-8. Therefore, larger prediction errors indicate a higher probability of misidentifying the severity levels and severity scores. According to the literature [5], treatment policies and care methods differ depending on the severity level of depression. Hence, the highly accurate prediction of the severity score is crucial. However, the existing models exhibited considerably large prediction errors; therefore, they were likely to output inaccurate prediction scores across different severity levels. Conversely, the proposed model considerably reduces the prediction error and thus can make it less likely to deviate from the correct severity level.

Since the existing models in Table 3 have not presented SMAPE, a comparison with the proposed model is not possible. However, SMAPE can provide one finding regarding the proposed model by considering the contents of Figure 2. As can be seen from the SMAPE equation, the prediction errors for smaller actual severity scores can strongly influence the increase in SMAPE. From Figure 2, a discrepancy between actual and predicted scores is observed for the small severity scores of approximately 0 to 5. Conversely, in Figure 2, no such large discrepancies in the distribution of actual and predicted scores are observed for the large severity scores. Thus, the value of SMAPE in the proposed model may reflect the prediction errors in the small severity scores.

As reported by Fang et al. [14], multimodal models can effectively improve the prediction accuracy of depression severity. Improving the prediction accuracy of the different modalities is critical for the further improvement of the performance of multimodal models. Table 3 shows that the audio modalities had the largest prediction errors compared with those of the other modalities. Improving the audio modality can considerably enhance the prediction accuracy of multimodal models. In this study, we significantly reduced the prediction error compared with those of the existing models. Therefore, this study will play a role in improving the prediction performance of multimodal models in the future.

For the practical use of the model, the following three points need to be considered. The first is that the same subject can be included in both the training data and test data in the prediction experiments, which were conducted in the same experimental setting as the existing studies. However, in actual medical practice, new patients not included in the training data can be also diagnosed. Therefore, it is necessary to evaluate the generalization performance for new patients. In the future, prediction experiments should be conducted in a setting where different subjects are divided between the training and test data. The second point is that the model requires numerous computational resources. The proposed model has seven network layers for processing training data. Therefore, there are a huge number of parameters, and using a computer with a high-performance GPU becomes essential. Actual medical practice requires a simpler model; hence, model compression techniques need to be implemented [25,26]. The third point is that the robustness of the model against noise needs to be improved. The voice data used in this experiment were recorded in a quiet environment using the same recording equipment at the designated location for all subjects. However, it is not always possible to record voices under such favorable conditions in actual medical settings. To make the proposed model practical in a wider range of applications, it is important to achieve accurate severity prediction using noisy speech recorded with inexpensive devices or via telephone or video calls. To achieve this objective, it is necessary to introduce a noise reduction process [27] in the preprocessing of speech data. The introduction of a noise reduction process is expected to enable noise-robust depression diagnosis support not only in a face-to-face format but also in a remote format.

The key benefits of the proposed model are summarized as follows:

▪ The proposed model can predict depression severity based on the correlations among audio features obtained from speech data.
▪ Despite the fact that the proposed model only uses speech data, it performs much better than the existing state-of-the-art models, including multimodal models.

The key limitations of the proposed model are listed as follows:

▪ A substantial amount of training data, high-performance computational resources, and a considerable amount of computational time are required for constructing the proposed model.
▪ The number of neighborhoods with strong correlations may differ for each audio feature. However, the proposed model has a limitation in that the number of neighbors is fixed.

## 5. Conclusions

In this paper, we proposed a new regression model based on GCNNs to predict depression severity scores. The proposed model enabled depression severity prediction based on the correlation among audio features, which was not considered previously. The experimental results demonstrated that the proposed model has an RMSE, MAE, and SMAPE of 2.15, 1.25, and 50.96%, respectively. Notably, the RMSE and MAE were considerably better than those of the current state-of-the-art prediction methods. Hence, the proposed model can be a promising support tool for the diagnosis of depression for medical as well as personal use. In the future, for practical use, we will evaluate the generalization performance of the model and introduce a model compression technique and a noise reduction process.

## Figures and Tables

**Table 1:** Correspondence between PHQ-8 score and severity level.

PHQ-8 Scores	0	1	2	3	4	5	6	7	8	9	10	11	12	13	14	15	16	17	18	19	20	21	22	23	24
Diagnosis	Non-depression										Depression														
Severity level	Nonsignificant					Mild					Moderate					Moderately severe					Severe				

**Table 2:** Details of the architecture of the proposed model.

Layer	Number of Kernels	Kernel Size	Dropout Rate	Activation Function
Graph_conv1	64	1 × 9 × 64	-	ReLU
Graph_conv2	128	64 × 9 × 128	-	ReLU
Graph_conv3	256	128 × 9 × 256	-	ReLU
Graph_conv4	512	256 × 9 × 512	-	ReLU
Dense1	-	-	0.1	tanh
Dense2	-	-	0.1	tanh
Dense3	-	-	0.1	tanh

**Table 3:** Prediction results and comparison with the existing methods.

Author	Year	Modality	Method	Feature	RMSE	MAE	SMAPE
Yang et al. [11]	2017	A	DCNN-DNN	GeMAPS based audio features	5.63	4.85	-
		V	DCNN-DNN	Histogram of Displacement Range	5.40	4.75	-
		T	DCNN-DNN	Paragraph Vector	4.38	3.64	-
		A + V + T	DCNN-DNN fusion framework	-	5.97	5.16	-
Yang et al. [12]	2020	A	DCNN	DCGAN generated features	5.52	4.63	-
Lu et al. [13]	2022	A	Transformer Encoder + CNN	eGeMAPS based audio features	5.37	4.48	-
Fang et al. [14]	2023	A	LSTM + FFN	COVAREP based audio features and Formant	6.13	5.21	-
		V	LSTM + FFN	3D facial_landmark, Head Pose, Action Units, Eye Gaze	5.44	4.12	-
		T	Bi-LSTM + Attention	USE Embedding	4.76	3.61	-
		A + V + T	Feature fusion network	-	3.68	3.18	-
Ours	2023	A	GCNN	65 audio features	2.15	1.25	50.96%

## Data Availability

Not applicable.
